# Cannabinoid Hyperemesis Syndrome: The challenge of diagnosis and management – A case report

**DOI:** 10.1192/j.eurpsy.2025.973

**Published:** 2025-08-26

**Authors:** C. P. Murta, F. Silva, L. Kobayashi

**Affiliations:** 1 Psychiatry, ULS Algarve, Faro, Portugal

## Abstract

**Introduction:**

Cannabinoid Hyperemesis Syndrome (CHS) is a condition associated with long-term cannabis use, marked by recurrent episodes of nausea, vomiting and abdominal pain, typically relieved by hot showers—a nearly pathognomonic feature. It often develops after years of heavy cannabis use, with symptoms recurring cyclically every few weeks to months while the individual continues to use cannabis. Symptom resolution is generally observed after cessation. Though cannabis is commonly used to relieve nausea (as in chemotherapy-induced vomiting), in susceptible individuals, prolonged use paradoxically induces these symptoms, presenting a diagnostic and therapeutic challenge. Recognizing CHS is increasingly important as global cannabis consumption rises.

**Objectives:**

The aim of this study is to review the clinical presentation of CHS, highlighting key diagnostic features, current management strategies and treatment.

**Methods:**

A comprehensive case report of a 27-year-old female with a history of heavy cannabis use was conducted. She presented with severe nausea and vomiting for two weeks following reported cannabis cessation. A thorough clinical evaluation was undertaken to better understand the clinical presentation of CHS. Additionally, a literature review was performed using PubMed to gather relevant clinical articles on CHS.

**Results:**

The patient exhibited hallmark features of CHS, including a prolonged history of cannabis use beginning in adolescence, recurrent episodes of severe nausea, vomiting, abdominal pain, the typical compulsive use of hot showers for symptom relief and lack of response to conventional antiemetic treatments. These features, combined with the patient’s lack of motivation to discontinue cannabis despite symptom recurrence, strongly support the diagnosis of CHS. Although she had a history of an eating disorder and presented with the Russell sign and dental damage consistent with chronic vomiting, no psychiatric comorbidities or body image disturbances were identified.

**Conclusions:**

CHS remains an under-recognized condition that poses diagnostic challenges. This case reinforces the need to inquire about cannabis use in patients presenting with cyclic vomiting, abdominal pain and weight loss. CHS symptoms and pathophysiological mechanisms can mimic other important diseases, such as eating disorders or gastrointestinal pathologies. Challenges in managing CHS include patient skepticism regarding the role of cannabis as a cause of symptoms, perceived benefits of cannabis and a lack of other effective therapies. Management of CHS involves both acute symptomatic treatment and long-term cessation of cannabis use. Acute interventions may include fluid resuscitation, haloperidol administration, and topical capsaicin application. However, the only definitive treatment is complete abstinence from cannabis, which leads to symptom resolution in the vast majority of cases.

**Disclosure of Interest:**

None Declared

